# New Insights into the Oenological Significance of *Candida zemplinina*: Impact of Selected Autochthonous Strains on the Volatile Profile of Apulian Wines

**DOI:** 10.3390/microorganisms8050628

**Published:** 2020-04-26

**Authors:** Pasquale Russo, Maria Tufariello, Raffaela Renna, Mariana Tristezza, Marco Taurino, Lorenzo Palombi, Vittorio Capozzi, Carlo G. Rizzello, Francesco Grieco

**Affiliations:** 1 Department of the Sciences of Agriculture, Food and Environment, University of Foggia, 71121 Foggia, Italy; 2 CNR—Institute of Sciences of Food Production (ISPA), via Prov.le, Lecce-Monteroni, 73100 Lecce, Italy; maria.tufariello@ispa.cnr.it (M.T.); marianatristezza@hotmail.com (M.T.); marco.taurino@ispa.cnr.it (M.T.); 3 Department of Soil, Plant and Food Science, University of Bari Aldo Moro, Via Amendola, 165/a, 70126 Bari, Italy; renna481@gmail.com (R.R.); carlogiuseppe.rizzello@uniba.it (C.G.R.); 4 CNR—Institute for Applied Physics ‘Nello Carrara” (IFAC), Via Madonna del Piano 10, Sesto Fiorentino, 50019 Firenze, Italy; l.palombi@ifac.cnr.it; 5 Institute of Sciences of Food Production, National Research Council (CNR), c/o CS-DAT, Via Michele Protano, 71121 Foggia, Italy; vittorio.capozzi@ispa.cnr.it

**Keywords:** *Candida zemplinina*, *Starmerella bacillaris*, yeast, biodiversity, wine, alcoholic fermentation, volatile organic compound

## Abstract

In this investigation, we explored the oenological significance of *Candida zemplinina* (syn. *Starmerella bacillaris*) isolates from Apulian grape musts. Moreover, we provide the first evidence of the impact of different *C. zemplinina* strains on the wine aromatic properties tested as monocultures. We described the diversity of *C. zemplinina* strains isolated from grapes and the variability of ‘volatile’ phenotypes associated with this intraspecific variability. Thirty-three isolates were characterized at strain level by PCR-based approach and, among these, 16 strains were identified and then tested by microfermentation tests carried out in grape must. Analyzed strains were low producers of acetic acid and hydrogen sulphide, not able to decarboxylate a panel of representative amino acids, whereas they showed fructophilic character and significant glycerol production. Volatile profiles of produced wines were investigated by gas chromatography–mass spectrometry. The Odor Activity Values of all molecules were calculated and 12 compounds showed values above their odor thresholds. Two selected strains (35NC1 and 15PR1) could be considered as possible starter cultures since they were able to positively affect the sensory properties of obtained wine. This report firstly supplies evidence on the strain-specific impact of different *C. zemplinina* strains on the final aroma of produced wines.

## 1. Introduction

The transformation of grape juice into wine is a complex microbial reaction characterized by the sequential development of various species and strains of oenological yeasts. Traditionally, the spontaneous fermentation process is driven by indigenous microbes associated with grapes and the winery environment [1,2,3]. Among these yeasts, the early stages of fermentation are characterized by progressive development of non*-Saccharomyces* yeasts, such as *Candida* spp., *Hanseniaspora* spp., *Kluyveromyces* spp., *Pichia* spp., and *Rhodotorula* spp., and subsequently, *S. cerevisiae* proliferates, dominating and completing the wine fermentation [4]. Generally, non*-Saccharomyces* yeasts were considered of secondary significance or undesirable to the process, but, in the last years, their important role in the fermentation process has been considered [4,5,6]. Recent studies have shown that non-*Saccharomyces* yeasts have different oenological properties compared to those of *S. cerevisiae*, and can be used to modulate and improve the aroma and complexity of wines [7,8,9,10]. In fact, when used in combination with *Saccharomyces* strains, these species are able to improve wine organoleptic quality and sensory notes [4,5,11]. Among non*-Saccharomyces* yeasts, *Torulaspora delbrueckii*, *Metschnikowia pulcherrima*, *Pichia kluyveri*, *Lachancea thermotolerans* are already commercialized as oenological starter cultures [12], while the oenological properties of other species, such as *Hanseniaspora uvarum* and *Candida zemplinina* (syn. *Starmerella bacillaris*), have been the subject of several studies [13,14,15]. Recent studies delved into the oenological significance of *H. uvarum* strains isolated from Apulian musts [16,17] but no information is available about the fermentative properties of *C. zemplinina* strains identified in the above conditions [18,19,20].

It was already shown that *C. zemplinina* strains may play relevant activities during winemaking, due to their remarkably fructophilic nature and low ethanol production rate [14,21]. Strains belonging to this species, when inoculated at first, were able to alleviate the osmotic stress of *S. cerevisiae* cells by selectively consuming sugars [22]. Several studies assessed the oenological significance of *C. zemplinina* strains employed in combination with *S. cerevisiae* [22,23,24,25]. Recently, Barbera wines produced with *C. zemplinina* and *S. cerevisiae* were characterized by higher amounts of glycerol and esters responsible for fruity notes [26]. Englezos and coworkers [26] demonstrated that the wines produced with mixed cultures of *C. zemplinina* and *S. cerevisiae* contained higher values of esters compared to wines fermented with *S. cerevisiae* alone. To the best of our knowledge, only two studies [24,27] evaluated the impact of *C. zemplinina* monoculture inoculation on wine volatiles and each of these studies tested only one strain. The novelty of the present investigation is mainly to increase the limited knowledge available about the specific impact of different *C. zemplinina* strains during the winemaking process.

The present study describes the genetic characterization of a collection of Apulian *C. zemplinina* isolates at the strain level. The identified strains were tested as monocultures in microfermentation trials monitoring the chemical and volatile profiles during the fermentative process, allowing an investigation of the strain-specific influence of the selected strains on produced volatile compounds.

## 2. Materials and Methods

### 2.1. Yeast Strains

Yeast strains used in the present study were deposited in Agro-Food Microbial Culture Collection of ISPA (http://www.ispacnr.it/collezioni-microbiche). Yeasts were cultured in YPD broth (10 g/L yeast extract, 20 g/L peptone, 20 g/L glucose, 20 g/L agar) at 28 °C for 24 h, and maintained at −80 °C in glycerol 50% [28]

### 2.2. Molecular Characterization

Total genomic DNA was extracted as previously described by Tufariello et al. [29]. The genetic analysis was carried out by using the following primers: R5 (5′-AACGCGCAAC-3′; T_m_ 38 °C), RF2 (5′-CGGCCCCTGT-3′; T_m_ 42 °C) [30], 1283 (5′-GCGATCCCCA-3′; T_m_ 42 °C), M13 (5′-GAGGGTGGCGGTTCT -3′; T_m_ 53 °C), (GTG)_5_ (5′-GTGGTGGTGGTGGTG -3′; T_m_ 53 °C) [31]. The reactions were carried out using Taq DNA polymerase (Thermo Scientific, Waltham, MA, USA) and the following parameters: 40 cycles of 1 min at 94 °C, 1 min at the primer-specific T_m_, 2 min at 72 °C, with a final cycle of 10 min at 72 °C. The amplicon profiles obtained in the above described PCR reactions were analyzed with the Gel Compar 3.1 software (Applied Math, Kortrijk, Belgium).

### 2.3. Microfermentations

The strain-specific fermentative properties of the identified strains were assessed by microfermentation in must from Negroamaro grapes (sugars 201 g/L, 21.1 °Brix, pH 3.4, assimilable nitrogen concentration 141.14 g/L). The must was firstly centrifuged (10 min at 8000× *g*) and then sterilized by membrane filtration (0.45 mm Ø membrane). Five hundred milliliters of treated must were aliquoted in sterile Erlenmeyer flasks and then inoculated with 10^6^ CFU/mL of *C. zemplinina* precultured in the same must, for 48 h at 25 °C. The kinetics of the fermentations were monitored daily by gravimetric determinations, recording the weight decrease caused by the release of CO_2_. When the sample reached a constant weight, they were directly processed or stored at –20 °C for further analysis. Each fermentation experiment was achieved by carrying out three simultaneous independent repetitions.

### 2.4. Technological Characterization

The qualitative hydrogen sulphide production was determined by the blackening of the PbAcO paper inserted between the plug and inner wall of the Erlenmeyer, above the level of the liquid. Based on the results obtained, the isolates were classified as high (+++), medium (++), low (+), and no (-) sulphide producers [32]. The identification of yeast strains keeping an amino acid (acids histidine, tyrosine, phenylalanine, tryptophan, lysine, leucine, and arginine) decarboxylation activity was assessed by a plate assay method [20].

### 2.5. Chemical Analysis

The chemical analysis of wines and musts was carried out by Fourier transform infrared spectroscopy (FTIR) using the WineScan Flex (FOSS Analytical, Hillerød, Denmark). Samples were firstly centrifuged at 8000× *g* for 10 min and then subjected to the analysis [33]. Extraction of volatile compounds in wines was carried out by means of solid-phase extraction (SPE), according to Fragasso et al. [34]. The chromatographic analysis was conducted as described by Tufariello et al. [35]. To 50 mL of each wine sample and each standard solution were added 300 µL of 2-octanol solution (100 mg/mL). The extraction of volatile compounds was carried out by adsorption/desorption on strata polymeric SPE sorbents (styrene–divinylbenzene) prepacked in 500 mg/6 mL cartridges (Phenomenex, Torrance, CA, USA). The analyses were performed in triplicate. The concentration of each volatile compound was expressed as μg internal standard equivalents/L wine, obtained by normalizing the compound peak area to that of the internal standard and multiplying by the concentration of the internal standard (2-octanol). The analysis of volatile wine compounds previously extracted by SPE method was performed by gas chromatography coupled to quadrupolar mass spectrometry, using an Agilent 5973 Network detector (Agilent Technologies, Palo Alto, CA, USA). Analytes were thermally desorbed at 250 °C, and then separated on an HP-INNOWAX capillary column (60 m × 0.25 mm, 0.25 μm, J&W Scientific Inc., Folsom, CA, USA). The elution program started at a temperature of 40 °C, which was held for 5 min, then increased to 220 °C at a rate of 3 °C/min; the final temperature was held for 15 min before returning to the initial value. The carrier gas (He) flow rate was 1 mL/min. The separated analytes were transferred into the ion source of the mass spectrometer, kept at 240 °C, through a transfer line, kept at 230 °C. Their detection was carried out by electron ionization mass spectrometry, operated in total ion current (TIC) mode, using ionization energy of 70 eV. The m/z acquisition range was 35–400. Data were collected with the HP Chemstation software (Agilent Technologies). Thirteen compounds were identified by comparing their retention times and mass spectra with those of pure compounds analyzed under the same conditions. For other extracted compounds, the comparison of MS fragmentation patterns with those included in the National Institute for Standards and Technology database (NIST 02, *p* > 80) was employed to achieve a tentative identification.

### 2.6. Statistical Analysis

Significant differences among samples were determined for each chemical compound by analysis of variance (post hoc Tukey, α = 0.05). Statistical data processing was performed using the free software package STATISTICA 7.0 software (StatSoft software package, Tulsa, OK, USA). Cluster analysis and microbial diversity indices calculation were performed using the free software package PAST [36]. Aroma profile data pretreatment (calculation of standardized concentrations), statistical analysis (Principal Component Analysis), and presentation of results were carried out using routines written in the MATLAB programming environment.

## 3. Results

### 3.1. Genetic, Molecular, and Technological Characterization of the C. zemplinina Strains

**Figure 1:**
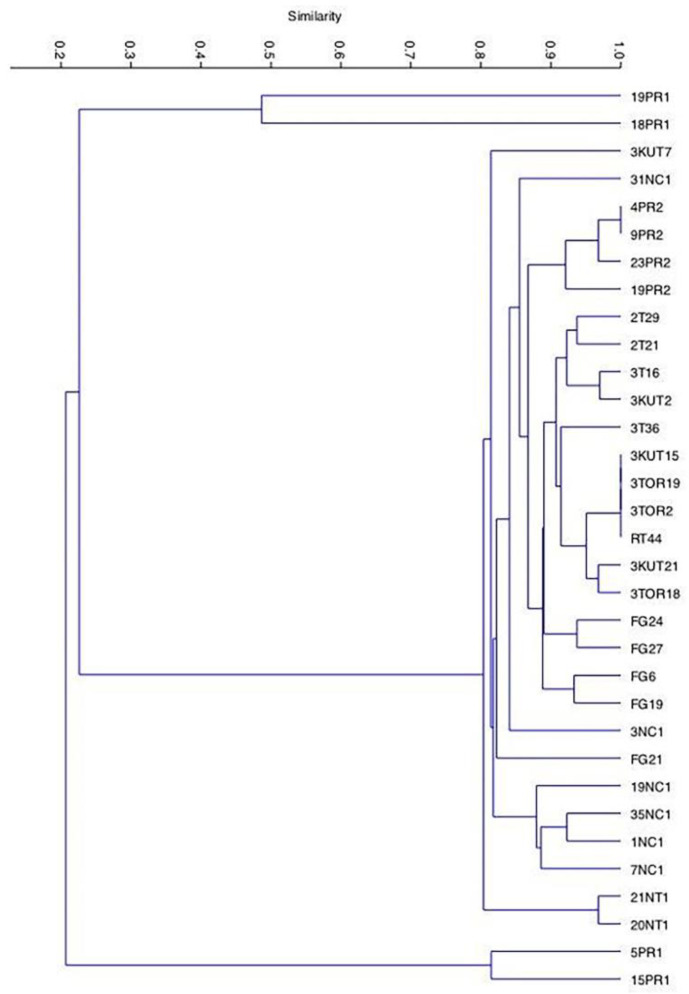
The Unweighted Pair Group Method with Arithmetic mean (UPGMA) dendrogram generated by cluster analysis of interdelta region patterns obtained from the 33 Apulian *C. zemplinina* strains. The percentage of similarity is indicated.

The analyzed population was composed of 33 *C. zemplinina* isolates (Table S1) selected from spontaneous fermentations of Negroamaro grape must and formerly characterized at the species level [20]. To assess the diversity within this population, the discerning capacity of three PCR-based methods were evaluated (i.e., Random Amplification of Polymorphic DNA (RAPD), minisatellites and microsatellites analysis) by separately using means of the following primers: (i) R5, R12, and 1283 (RAPD-PCR; Figures S1–S3); (ii) M13 (minisatellite sequences evaluation; Figure S4); (iii) (GTG)_5_ (macrosatellite sequences analysis; Figure S5). The dendrogram analysis of the data set was based on the presence or absence of major bands produced by the above five primers. The statistical analysis of the obtained data allowed the identification in each population of three distinct strain clusters with a similarity value within 90%. The obtained clustering indicated that the analyzed population was composed of 16 different strains, thus showing a biodiversity value equal to 80%, within the population itself (Figure 1). The technological and oenological properties of the above-selected strains (7NC1, 35NC1, 19NC1, 31NC1, 3NC1, 23PR2, 20NT1, 5PR1, 15PR1, 19PR1, 18PR1, 3T16, 3KUT7, FG6, FG21, FG27) were then assessed by microfermentative assays in order to evaluate properties of oenological interest.

All the analyzed strains showed the same fermentation kinetics characterized by the start of the alcoholic fermentation process, 48 h after inoculation (Figure S6). The qualitative analysis of the microfermentations showed a reduced production of H_2_S for all strains. Hydrogen sulphide, a secondary metabolite in the synthesis of sulphur amino acids and produced in fermentation by yeasts, is an undesirable compound because it causes a rotten egg odor. The ability of yeasts to produce biogenic amines by decarboxylation of the corresponding amino acid (His, Tyr, Phe, Trp, Lys, Leu, and Arg) has been determined (Table S2). According to data obtained, the 19NC1 and 18PR1 strains were able to decarboxylate arginine, whereas the FG6 and FG27 strains showed decarboxylation of tyrosine. All the other strains showed no activity on the amino acids tested. The results of the chemical analysis of the produced wines are shown in Table 1.

We found ethanol content varying between 8.24% and 10.44%. All the strains produced a significant amount of glycerol, whose concentration ranged from 8.80 to 9.98 g/L in the obtained wines. Concerning acetic acid production expressed as volatile acidity, no values higher than 0.6 g/L were detected and, in particular, the 35NC1 showed the lowest production (0.35 g/L). All 16 *C. zemplinina* strains showed preferential consumption of fructose.

### 3.2. Analysis of Volatile Compounds

The strain-specific aptitude to produce volatile compounds involved in the wine flavor was evaluated and the results are shown in Table 2 and Table 3. Twenty-four volatile molecules were identified and quantified in wines, including alcohols, esters, terpenes, volatile phenols and acids, lactones, and norisoprenoids.

Alcohols represented the major group for all the wines with concentrations ranging from 55.83 mg/L (7NC1) to 131.06 mg/L (35NC1) followed by esters and terpenes. In particular, 3-methyl-1-butanol and phenylethanol were detected at higher concentrations in all samples ranging from 24 mg/L (3T16) to 65.83 mg/L (35NC1) and from 11.97 mg/L (7NC1) to 46.69 mg/L (35NC1), respectively. The Odor Activity Values (OAVs), a marker of the influence on wine aroma of individual volatile molecules, were calculated by dividing the mean concentration of each compound by its odor threshold value (OTH) as previously described [35,36]. Table 4 and Table 5 show the molecules with OAV > 1 considered as odor-active compounds.

The 3-methyl-1-butanol and phenylethanol are the molecules with OAV > 1 contributing fine fruity and rose odor to the wines [35]. 1-Hexanol, 3-hexen-1-ol cis (Z) and trans (E), and 2-methyl-1-propanol were also detected, but at amounts singularly low for their impact on the wine aroma (OAVs < 1). Regarding esters, the concentrations revealed ranged from 0.34 mg/L in wine fermented by FG27 strain to 7.99 mg/L in wine added with 35NC1 strain. The ethyl esters of fatty acids (ethyl octanoate and decanoate) showed important variations in their concentrations (Table 2 and Table 3) which were wider for ethyl decanoate (0.76 mg/L–6.70 mg/L). As shown in Table 4, ethyl decanoate exhibits an OAV > 8 in all samples except FG27, FG6, and 15PR1, susceptible to imparting notes of fruitiness and sweetness to these wines. Regarding odor activity, the second most representative ester was ethyl octanoate revealed in concentrations above its odor threshold in wines fermented by 35NC1, 3KUT7, 31NC1, and FG6. Fatty acids have been described as giving a general rise to fruity, cheesy, fatty, and rancid notes [35]. In our case, we detected two acids, methyl butanoic and methyl propanoic acids, in concentrations below their odor threshold, except in wines produced by 31NC1 and FG21 characterized by 3-methylbutanoic acid at values above its odor threshold (Table 4 and Table 5). The norisoprenoid β-damascenone was found in amounts ranging from 0.08 to 0.75mg/L in the wines produced with 35NC1, 3KUT7, FG21, FG27B, 5PR1, and 19NC1. This compound is related to flowery, sweet, and fruity notes, and its concentration in all samples was above its odor threshold. Finally, six terpenes were detected in wines. They were linalool, α-terpineol, citronellol, geraniol, ho-trienol (42 μg/L), and [*E*,*E*]-farnesol. Their concentrations were low, but linalool, geraniol, and citronellal had OAVs higher than one.

**Figure 2:**
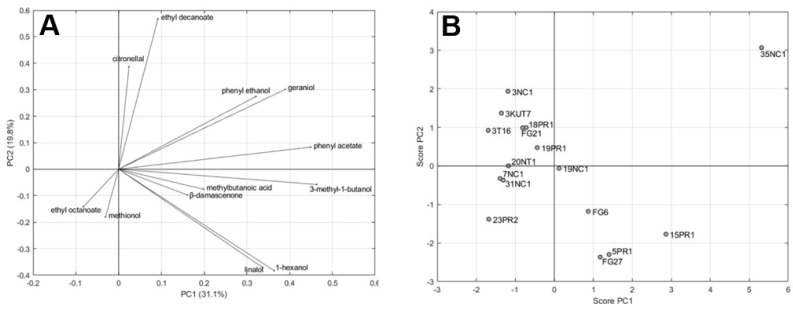
Two-dimensional principal component analysis (PCA). Loading plot (**A**) for volatiles having OAV > 1 and scores plot (**B**) for selected strains as variables.

In order to understand the variability among the strains and the impact on the aroma profile, the volatile compounds present at concentrations above their odor threshold (Table 4 and Table 5) were submitted to the principal component analysis (PCA; Figure 2). For each molecule, standardized concentrations were obtained by scaling absolute concentrations to the same sample mean (equal to zero) and sample standard deviation (equal to one).

Figure 2A shows the loadings plot of the first two PCs, which jointly accounted for 50.9% of the total variance (31.1% and 19.8% by PC1 and PC2, respectively), used to establish the relative importance of each volatile component in order to relate volatile compounds with positive odor impact to one another and with samples. Figure 2B shows the scores scatter plot of the 16 samples.

The loading plot shows that almost all the volatiles are located in the first and fourth plane (positive PC1 values). This allows discriminating in the corresponding score plot the samples with an overall volatile concentration below the average (3NC1, 3KUT7, 3T16, FG21, 18PR1, 19PR1, 20NT1, 7NC1, 31NC1, and 23PR2) and above the average (19NC1, FG6, FG27, 5PR1, 15PR1, and 35NC1). This statistical representation confirmed strong oenological similarities shown by strains 3KUT7 and FG21. The wines made inoculating these two biotypes presented very close levels of ethanol and sugars (Table 1), and behaved similarly when aroma compounds were considered (Table 2; Figure 2).

The loading plot also suggests that PC2 can be used to highlight a different aromatic profile of the samples. Positive values of PC2 correspond to relatively high concentrations of certain compounds such as citronellol, etyl decanoate, phenyl ethanol, and gerianol. Negative values correspond to relatively higher concentrations of compounds such as linalol, 1-hexanol, ethyl octanate, and methionol.

On the whole, the analysis of the scatter plot allows highlighting two samples (35NC1 and 15PR1) that differ from the others in their relatively higher overall concentration of the compounds and from each other in their aromatic profile.

**Figure 3:**
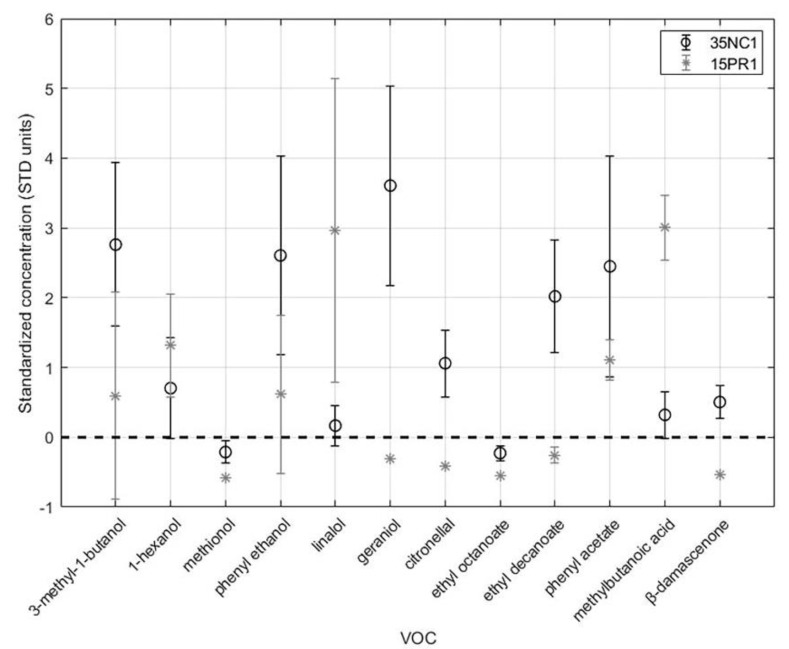
Comparison of standardized concentrations of volatile compounds having OAV > 1 in wines produced with 35NC1 and 15PR1B strains.

A significant presence of geraniol, phenylethanol, phenyl acetate, ethyl decanoate, and citronellol positively characterized the 35NC1 sample, while higher values of 3-methyl-1-butanol, methylbutanoic acid, β-damascenone, 1-hexanol, and linalool distinguished wines fermented with the 15PR1 strain. The statistical validation allowed identifying the above two strains as significant producers of important aroma-contributing compounds (OAV > 1). The standardized concentrations of these two samples are compared in Figure 3. For each standardized concentration, the error bars are representative of the standard deviation obtained by considering the six replicates.

## 4. Discussion

The interest in the use of autochthonous non*-Saccharomyces* strains is growing thanks to their valuable contribution to the fermentation process, the possibility to valorize specific grape musts, and the preservation of biodiversity in particular geographical areas, thus strengthening the terroir concept [37,38,39]. The main aim of this work was to broaden knowledge about the fermentation performance of *C. zemplinina* strains. In particular, for the first time, the production of volatile compounds in wines produced by monocultures of different strains belonging to this species was described.

Concerning the chemical composition, the absence of residual fructose confirmed the fructophilic character of *C. zemplinina* [21,40]. The production of relevant quantities of glycerol and low amounts of acetic acid was also observed, in agreement with previous studies [21,30]. Within the *Candida* genus, *C. stellata* and *C. versatilis* have been reported to be able to produce biogenic amines in wine and in soy sauce, respectively [41]. However, most of the strains considered did not show amino acid decarboxylation. Regarding the ‘volatile’ phenotype, alcohols and esters were the most representative compounds in all the wines’ volatile profiles and are produced by yeasts during the fermentative process. Among alcohols, 3-methyl-1-butanol and phenylethanol represented more than 80% of the alcoholic fraction. Overall, concentrations below 300 mg/L of these alcohols can have a positive impact on the wine, by conferring fruity and floral notes [42]. Under the conditions of this study, the wines produced by all the *C. zemplinina* monocultures did not exceed this concentration. We found a strain-specific release for several aroma compounds belonging to eight different families such as alcohols, esters, terpenes, acids, phenols, lactones, and furanic compounds. In particular, monoculture trial confirmed the strain-specific variability in higher alcohols, esters, fatty acids, terpenes, and carbonyl compounds reported by Binati et al. [23]. In addition, results produced the first evidence of a strain-specific production of norisoprenoids and lactones. Aware of the pros and the cons of Odor Activity Values (OAVs) as an indicator of the potential aroma contribution of individual compounds [36], we found that 12 compounds showed values above their odor thresholds. Among these, for the first time, we demonstrated (i) moderate, (ii) consistent, and (iii) high strain-dependent variability for (i) 3-methyl-1-butanol, (ii) 1-hexanol, phenylethanol, phenyl acetate, methyl butanoic acid, and (iii) methyonol, linalool, geraniol, citronellol, ethyl octanoate, ethyl decanoate, β-damascenone, respectively. It is important to highlight that these 12 compounds are only a part of the complex variability reported for volatiles. The reported findings provide phenotypical confirmation of the high genetic diversity depicted in winemaking environments for *C. zemplinina* [14,31] and, more in general, of the intraspecific variability of non-*Saccharomyces* in terms of oenological significance [4,5]. It is conceivable that phenotypic variability depends on both genetic properties of different strains and the influence of environmental factors [43]. In this regard, it is interesting to highlight that the similarity found in terms of oenological properties between strains genotypically distant, as it was the case of the two strains 3KUT7 and FG21, underlined the importance of studying the genetic basis associated with natural variation in oenological traits in *C. zemplinina* (as already reported in the model species *S. cerevisiae* [44,45]).

Considering geographical interest, it is important to underline that Apulia (Southern Italy) is the second region in Italy for wine production, particularly relevant for red and rosé wines [46]. Several studies delved into the study and the characterization of microbial diversity associated with grapes and wine fermentations [33,47,48]. Reporting the first study on a *C. zemplinina* population isolated from grapes/wines in the Apulian region, this investigation provides further insights into the Apulian microbiological diversity of oenological significance. The potential future perspectives of the present work mainly include the evaluation of the selected strains (i) at the industrial scale of wine production, (ii) in combination with *S. cerevisiae* strains, (iii) in terms of compatibility with malolactic starter cultures, (iv) as candidate starter cultures to produce alcoholic beverages from fruits other than grapevines [16,29,49,50,51,52,53,54].

This investigation, for the first time, highlights the strain-specific property of different *C. zemplinina* strains to modulate the volatile profile of the produced wine. Studies are now under way where the two selected strains, 35NC1 and 15PR1, will be tested as starter cultures for the industrial-scale production of regional typical wines.

## Figures and Tables

**Table 1:** Mean concentrations (g/L) and standard deviations (SD) of main chemical parameters in wines fermented by *C. zemplinina* strains.

ID	Ethanol (%v/v)	Sugars (g/L)	Glucose (g/L)	Fructose (g/L)	TA ^1^ (g/L)	VA ^2^ (g/L)	Tartaric (g/L)	Glycerol (g/L)	Malic (g/L)
7NC1	9.75 ± 1.16	51.44 ± 2.16	49.64 ± 7.17	0	5.20 ± 1.34	0.45 ± 0.11	1.33 ± 0.45	9.20 ± 1.10	1.95 ± 0.37
3NC1	10.13 ± 1.00	46.43 ± 2.94	44.37 ± 6.70	0	5.27 ± 1.95	0.44 ± 0.15	1.27 ± 0.14	9.58 ± 1.76	1.83 ± 0.55
19NC1	9.64 ± 1.80	53.89 ± 2.12	52.04 ± 5.21	0	5.26 ± 1.77	0.59 ± 0.17	1.52 ± 0.16	9.21 ± 1.11	1.91 ± 0.87
18PR1	9.75 ± 1.90	51.70 ± 2.80	49.22 ± 7.10	0	5.42 ± 1.73	0.58 ± 0.14	1.36 ± 0.21	9.46 ± 1.15	2.03 ± 0.94
FG21	10.44 ± 1.26	39.62 ± 2.27	36.98 ± 4.56	0	5.40 ± 1.73	0.45 ± 0.14	1.22 ± 0.20	9.42 ± 1.77	1.97 ± 0.88
20NT1	9.98 ± 1.36	46.52 ± 1.70	43.85 ± 5.10	0	5.45 ± 1.55	0.41 ± 0.11	1.22 ± 0.24	9.97 ± 1.11	1.96 ± 0.76
19PR1	8.95 ± 1.77	63.45± 2.77	61.7 ± 7.28	0	5.66 ± 1.84	0.54 ± 0.13	1.65 ± 0.35	9.51 ± 1.78	2.13 ± 0.58
3KUT	10.32 ± 1.95	39.32 ± 2.11	36.77 ± 4.37	0	5.28 ± 2.11	0.43 ± 0.17	1.23 ± 0.24	9.51 ± 1.61	1.80 ± 0.91
35NC1	10.31 ± 1.10	43.35 ± 2.73	40.84 ± 4.67	0	5.25 ± 1.95	0.35 ± 0.15	1.22 ± 0.35	9.90 ± 1.70	1.91 ± 0.75
31NC1	9.89 ± 1.65	48.96 ± 2.10	46.84 ± 6.20	0	5.52 ± 1.77	0.45 ± 0.18	1.31 ± 0.25	9.97 ± 1.16	1.95 ± 0.81
23PR2	9.62 ± 1.88	53.00 ± 3.00	50.82 ± 5.66	0	5.21 ± 1.94	0.54 ± 0.16	1.32 ± 0.45	9.42 ± 1.74	1.99 ± 0.92
FG27	8.24 ± 1.94	75.86 ± 2.26	74.28 ± 7.10	0	5.55 ± 1.76	0.52 ± 0.16	1.84 ± 0.61	9.59 ± 1.44	2.07 ± 0.95
FG6	10.36 ± 1.10	41.58 ± 2.75	40.60 ± 5.48	0	5.45 ± 1.63	0.43 ± 0.14	1.32 ± 0.21	8.80 ± 1.70	2.03 ± 0.33
5PR1	9.70 ± 1.74	52.21 ± 2.38	50.35 ± 4.90	0	5.34 ± 1.80	0.41 ± 0.16	1.25 ± 0.43	9.37 ± 1.16	2.09 ± 0.51
15PR1	8.81 ± 1.81	66.12 ± 2.77	64.02 ± 7.21	0	5.56 ± 1.93	0.47 ± 0.21	1.45 ± 0.24	9.98 ± 1.96	2.10 ± 0.43
3T16	10.10 ± 1.17	46.02 ± 1.96	44.19 ± 5.10	0	5.44 ± 2.10	0.41 ± 0.20	1.32 ± 0.25	9.25 ± 2.77	2.09 ± 0.46

Values are the mean of two injections of each replicate (𝑛 = 6); the standard deviation values (±) are indicated. **^1^**TA, total acidity; **^2^**VA, volatile acidity. No statistical differences (one-way Anova *p* < 0.05) were revealed.

**Table 2:** Volatile compounds identified in wines fermented by the indicated *C. zemplinina* strains.

Compounds	7NC1	3NC1	35NC1	3KUT7	19PR1	31NC1	FG21	FG27
Alcohols								
2-Methyl-1-propanol	6.55 ± 1.46	4.82 ± 0.84	15.83 ± 4.76	7.18 ± 1.87	6.73 ± 2.55	11.18 ± 5.85	7.82 ± 2.55	4.045 ± 0.94
3-Methyl-1-butanol	35.59 ± 5.10	35.16 ± 5.27	65.83 ± 11.80	26.02 ± 4.52	34.35 ± 5.79	38 ± 7	30.46 ± 7.38	48.57 ± 7.90
1-Hexanol	0.83 ± 0.11	0.73 ± 0.33	1.50 ± 0.44	0.56 ± 0.11	0.67 ± 0.26	0.87 ± 0.45	0.73 ± 0.22	1.8 ± 0.25
3-Hexen-1-ol (Z)	nd ^1^	nd	0.18 ± 0.04	nd	nd	nd	nd	nd
3-Hexen-1-ol (E)	0.16 ± 0.05	0.11 ± 0.04	0.12 ± 0.05	nd	nd	nd	nd	0.43 ± 0.08
Methyonol	0.18 ± 0.06	0.22 ± 0.12	0.6 ± 0.26	nd	0.22 ± 0.05	0.46 ± 0.07	0.2 ± 0.05	1.76 ± 0.11
Benzylic alcohol	0.55 ± 0.14	0.06 ± 0.02	0.31 ± 0.06	0.47 ± 0.27	1.66 ± 0.47	0.32 ± 0.14	0.08 ± 0.02	0.60 ± 0.24
Phenylethanol	11.97 ± 6.70	18.28 ± 6.77	46.69 ± 11.79	23.92 ± 6.05	19.65 ± 6.74	15.08 ± 5.65	26.25 ± 6.17	17.6 ± 4.5
	**55.83 ± 13.62**	**59.38 ± 13.39**	**131.06 ± 29.20**	**58.15 ± 12.82**	**63.28 ± 15.86**	**65.91 ± 19.16**	**65.54 ± 16.39**	**74.80 ± 14.02**
Esters								
Ethyl lactate	nd	nd	0.04 ± 0.02	nd	nd	nd	nd	nd
Ethyl octanoate	nd	nd	0.03 ± 0.01	0.10 ± 0.02	nd	0.2 ± 0.04	nd	nd
Diethyl succinate	0.060 ± 0.02	0.30 ± 0.05	0.44 ± 0.15	0.18 ± 0.05	1.48 ± 0.34	0.4 ± 0.06	nd	nd
Ethyl decanoate	1.67 ± 0.33	3.86 ± 0.66	6.7 ± 1.65	4.05 ± 0.46	2.94 ± 0.44	3.5 ± 0.55	4.95 ± 0.94	nd
Phenyl acetate	0.20 ± 0.05	0.16 ± 0.06	0.78 ± 0.33	0.20 ± 0.07	0.55 ± 0.11	nd	0.15 ± 0.06	0.34 ± 0.07
	**1.93 ± 0.40**	**4.32 ± 0.77**	**7.99 ± 2.16**	**4.53 ± 0.60**	**4.97 ± 0.89**	**4.1 ± 0.65**	**5.1 ± 1.00**	**0.34 ± 0.07**
Terpenes								
Linalool	0.10 ± 0.04	0.11 ± 0.03	0.3 ± 0.07	0.14 ± 0.05	0.15 ± 0.05	0.16 ± 0.05	0.12 ± 0.04	0.6 ± 0.17
α- Terpineol	nd	nd	1.76 ± 0.65	nd	nd	nd	0.07 ± 0.03	0.83 ± 0.33
Geraniol	nd	nd	1.18 ± 0.43	nd	nd	nd	nd	nd
Citronellol	nd	0.15 ± 0.04	0.062 ± 0.02	0.07 ± 0.02	nd	nd	nd	nd
HO-Trienol	nd	0.55 ± 0.08	0.61 ± 0.20	nd	nd	nd	nd	nd
*trans* Farnesol	nd	nd	0.41 ± 0.11	nd	nd	nd	nd	nd
	**0.10 ± 0.04**	**24.81 ± 4.45**	**35.612 ± 6.66**	**0.21 ± 0.07**	**0.15 ± 0.05**	**0.16 ± 0.05**	**0.19 ± 0.07**	**1.43 ± 0.50**
Lactones								
Butyrolactone	0.11 ± 0.070	0.096 ± 0.011	0.15 ± 0.050	nd	nd	nd	0.11 ± 0.040	2.16 ± 0.11
Acids								
2-Methylpropanoic acid	nd	0.12 ± 0.04	0.32 ± 0.07	0.09 ± 0.03	0.087 ± 0.02	0.33 ± 0.070	0.13 ± 0.060	1.42 ± 0.17
Methylbutanoic acid	0.087 ± 0.011	nd	0.22 ± 0.08	0.07 ± 0.04	nd	0.42 ± 0.060	0.32 ± 0.070	nd
	**0.087 ± 0.011**	**0.12 ± 0.040**	**0.54 ± 0.150**	**0.16 ± 0.070**	**0.087 ± 0.02**	**0.75 ± 0.130**	**0.45 ± 0.130**	**1.42 ± 0.170**
Norisoprenoids								
β-Damascenone	nd	nd	0.22 ± 0.05	0.09 ± 0.01	nd	nd	0.08 ± 0.02	0.45 ± 0.07
Furaneol	nd	0.32 ± 0.06	0.45 ± 0.08	0.08 ± 0.02	nd	nd	0.34 ± 0.08	0.55 ± 0.05
		**0.32 ± 0.06**	**0.67 ± 0.13**	**0.17 ± 0.03**			**0.42 ± 0.10**	**1.00 ± 0.12**

Values are expressed in mg/L. Values are the mean of extractions of each sample; the standard deviation values (±) are indicated. **^1^**nd: not detectable.

**Table 3:** Volatile compounds identified in wines fermented by the indicated *C. zemplinina* strains.

Compounds	FG6	5PR1	23PR2	19NC1	20NT1	15PR1	18PR1	3T16
Alcohols								
2-Methyl-1-propanol	3.86 ± 0.77	5.8 ± 0.94	9.04 ± 2.75	1.34 ± 0.23	4.9 ± 0.95	5.44 ± 0.76	6.20 ± 1.67	7.12 ± 1.76
3-Methyl-1-butanol	45.03 ± 6.75	45.44 ± 11.65	35.16 ± 6.74	33.91 ± 7.10	34.32 ± 7.25	44 ± 15	31.9 ± 5.07	24 ± 6
1-Hexanol	1.67 ± 0.44	2.55 ± 0.88	0.85 ± 0.16	0.76 ± 0.44	0.63 ± 0.44	1.87 ± 0.45	0.71 ± 0.12	0.46 ± 0.12
3-Hexen-1-ol (Z)	nd ^1^	nd	5.9 ± 0.95	2.66 ± 0.45	nd	nd	nd	nd
3-Hexen-1-ol (E)	0.30 ± 0.06	nd	0.44 ± 0.07	nd	nd	0.51 ± 0.07	nd	0.04 ± 0.02
Methyonol	0.425 ± 0.17	0.66 ± 0.45	4.8 ± 0.94	5.25 ± 0.95	0.2 ± 0.06	nd	nd	0.212 ± 0.06
Benzylic alcohol	0.45 ± 0.25	0.65 ± 0.15	0.4 ± 0.07	0.11 ± 0.04	nd	0.37 ± 0.05	0.26 ± 0.12	0.075 ± 0.03
Phenylethanol	26.35 ± 5.38	21.51 ± 7.10	25.33 ± 5.17	33.44 ± 7.15	26.81 ± 5.11	30.22 ± 9.35	30.73 ± 6.77	28.09 ± 12.05
	**78.08 ± 13.82**	**76.61 ± 21.17**	**81.92 ± 15.84**	**77.47 ± 16.36**	**66.86 ± 13.81**	**82.41 ± 25.68**	**69.80 ± 13.75**	**59.99 ± 20.04**
Esters								
Ethyl octanoate	0.25	nd	0.24 ± 0.06	nd	nd	nd	nd	nd
Diethyl succinate	nd	0.3 ± 0.05	0.45 ± 0.11	nd	nd	nd	0.06 ± 0.02	0.08 ± 0.02
Ethyl decanoate	nd	nd	nd	3.55 ± 0.85	0.76 ± 0.24	2.06 ± 0.23	4.22 ± 0.37	3.11 ± 0.56
Phenyl acetate	0.4 ± 0.05	0.37 ± 0.06	nd	0.2 ± 0.06	0.15 ± 0.06	0.50 ± 0.06	0.2 ± 0.06	0.13 ± 0.04
	**0.65 ± 0.05**	**0.67 ± 0.11**	**0.69 ± 0.17**	**3.75 ± 0.91**	**0.91 ± 0.30**	**2.56 ± 0.29**	**4.48 ± 0.45**	**3.32 ± 0.62**
Terpenes								
Linalool	0.3 ± 0.07	0.5 ± 0.22	0.16 ± 0.05	0.132 ± 0.070	0.13 ± 0.05	0.97 ± 0.52	0.20 ± 0.05	0.09 ± 0.03
α- Terpineol	0.54 ± 0.11	0.48 ± 0.16	0.52 ± 0.11	0.55 ± 0.11	0.3 ± 0.05	0.11 ± 0.05	0.4 ± 0.06	0.33 ± 0.08
Geraniol	0.33 ± 0.07	nd	nd	nd	nd		nd	nd
*trans* Farnesol	nd	nd	0.65 ± 0.23	0.42 ± 0.05	0.34 ± 0.08	1.11 ± 0.27	0.458 ± 0.14	nd
	**1.17 ± 0.25**	**0.98 ± 0.38**	**1.33 ± 0.39**	**1.11 ± 0.23**	**0.78 ± 0.18**	**2.19 ± 0.84**	**1.058 ± 0.25**	**0.42 ± 0.11**
Lactones								
Butyrolactone	0.20 ± 0.050	0.8 ± 0.140	0.55 ± 0.10	0.09 ± 0.02	0.12 ± 0.07	0.4 ± 0.06	0.15 ± 0.04	nd
Acids								
2-Methylpropanoic acid	nd	nd	0.76 ± 0.15	0.18 ± 0.05	0.11 ± 0.04	0.58 ± 0.21	nd	0.07 ± 0.02
Methylbutanoic acid	nd	nd	nd	0.32 ± 0.08	nd	0.86 ± 0.11	0.24 ± 0.05	nd
	**0.000**	**0.000**	**0.76 ± 0.15**	**0.50 ± 0.13**	**0.11 ± 0.040**	**1.44 ± 0.32**	**0.24 ± 0.05**	**0.07 ± 0.02**
Norisoprenoids								
β-Damascenone	nd	0.22 ± 0.07	nd	0.75 ± 0.16	nd	nd	nd	nd
Furaneol	0.4 ± 0.05	0.93 ± 0.35	nd	nd	nd	nd	nd	nd
	**0.40 ± 0.05**	**1.15 ± 0.42**		**0.75 ± 0.16**				

Values are expressed in mg/L. Values are the mean of extractions of each sample; the standard deviation values (±) are indicated. **^1^** nd: not detectable.

**Table 4:** Odor threshold (Cs) and odor activity values (OAVs) of wines fermented by the *C. zemplinina* strains.

Compounds	C_S mg/L_	7NC1	3 NC1	35NC1	3 KUT7	19PR1	31NC1	FG21	FG27
3-Methyl-1-butanol	30	1.19	1.17	2.19	0.87	1.15	1.27	1.02	1.62
1-Hexanol	1.3	0.64	0.56	1.15	0.43	0.52	0.67	0.56	1.38
Methyonol	1.5	0.12	0.15	0.40	nd	0.15	0.31	0.13	1.17
Phenylethanol	10	1.20	1.83	4.67	2.39	1.97	1.51	2.63	1.76
Linalool	0.05	2.00	2.20	6.00	2.80	3.00	3.20	2.40	12.00
Geraniol	0.03	nd ^1^	nd	39.33	nd	nd	nd	nd	nd
Citronellol	0.018	nd	8.33	3.44	3.89	nd	nd	nd	nd
Ethyl octanoate	0.005	nd	nd	6.00	20.00	nd	40.00	nd	nd
Ethyl decanoate	0.2	8.35	19.30	33.50	20.25	14.70	17.50	24.75	nd
Phenyl acetate	0.25	0.80	0.64	3.12	0.80	2.20	nd	0.60	1.36
Methylbutanoic acid	0.25	0.35	nd	0.88	0.28	nd	1.68	1.28	nd
β-Damascenone	0.00005	nd	nd	4400.00	1860.00	nd	nd	1600.00	9000.00

Odor perception thresholds (mg/L) are reported in the literature by Tufariello et al. [35] and Capone et al. [36]. **^1^** nd: not detectable.

**Table 5:** Odor threshold (Cs) and odor activity values (OAVs) of wines fermented by the *C. zemplinina* strains.

Compounds	C_S mg/L_	FG6	5PR1	23PR2	19NC1	20NT1	15PR1	18PR1	3T16
3-Methyl-1-butanol	30	1.50	1.51	1.17	1.13	1.14	1.47	1.06	0.80
1-Hexanol	1.3	1.28	1.96	0.65	0.58	0.48	1.44	0.55	0.35
Methyonol	1.5	0.28	0.44	3.20	3.50	0.14	0.00	0.00	0.14
Phenylethanol	10	2.64	2.15	2.53	3.34	2.68	3.02	3.07	2.81
Linalool	0.05	6.00	10.00	3.20	2.64	2.60	19.40	4.00	1.80
Geraniol	0.03	11.00	nd ^1^	nd	nd	nd	nd	nd	nd
Citronellol	0.018	nd	nd	nd	nd	nd	nd	nd	nd
Ethyl octanoate	0.005	49.28	nd	48.00	nd	nd	nd	nd	nd
Ethyl decanoate	0.2	nd	nd	nd	17.75	3.80	10.30	21.10	15.55
Phenyl acetate	0.25	1.60	1.48	nd	0.80	0.60	2.00	0.80	0.53
Methylbutanoic acid	0.25	nd	nd	nd	1.28	nd	3.44	nd	nd
β-Damascenone	0.00005	nd	4400.00	nd	15,000.00	nd	nd	nd	nd

Odor perception thresholds (mg/L) are reported in the literature by Tufariello et al. [35] and Capone et al. [36]. **^1^** nd: not detectable.

## Supplementary Materials

The following are available online at https://www.mdpi.com/2076-2607/8/5/628/s1.
