# Supplementary material for: New Insights into the Oenological Significance of Candida zemplinina: Impact of Selected Autochthonous Strains on the Volatile Profile of Apulian Wines
Source: Microorganisms. 2020 Apr 26;8(5):628. doi: 10.3390/microorganisms8050628 (PMC7285007; doi:10.3390/microorganisms8050628)
Supplement: Supplementary file 1 [file microorganisms-08-00628-s001.zip › Table S1.docx]

Table S1: Selected yeasts used in this study

| **Nr** | **Isolate name** | **Vine** |  | **Nr** | **Isolate name** | **Vine** |
| --- | --- | --- | --- | --- | --- | --- |
| 1 | 1NC1 | Negroamaro |  | 17 | 2T29 | Negroamaro |
| 2 | 7NC1 | Negroamaro |  | 18 | 2T21 | Negroamaro |
| 3 | 35NC1 | Negroamaro |  | 19 | 3T36 | Negroamaro |
| 4 | 19NC1 | Negroamaro |  | 20 | 3T16 | Negroamaro |
| 5 | 31 NC1 | Negroamaro |  | 21 | 3KUT15 | Primitivo |
| 6 | 3NC1 | Negroamaro |  | 22 | 3KUT2 | Primitivo |
| 7 | 4PR2 | Primitivo |  | 23 | 3KUT21 | Primitivo |
| 8 | 9PR2 | Primitivo |  | 24 | 3KUT7 | Primitivo |
| 9 | 19PR2 | Primitivo |  | 25 | 3TOR19 | Primitivo |
| 10 | 23PR2 | Primitivo |  | 26 | 3TOR2 | Primitivo |
| 11 | 21NT1 | Nero di Troia |  | 27 | 3TOR18 | Primitivo |
| 12 | 20NT1 | Nero di Troia |  | 28 | FG6 | Nero di Troia |
| 13 | 5PR1 | Primitivo |  | 29 | FG19 | Nero di Troia |
| 14 | 15PR1 | Primitivo |  | 30 | FG21 | Nero di Troia |
| 15 | 19PR1 | Primitivo |  | 31 | FG24 | Nero di Troia |
| 16 | 18PR1 | Primitivo |  | 32 | FG27 | Nero di Troia |
