# Supplementary material for: New Insights into the Oenological Significance of Candida zemplinina: Impact of Selected Autochthonous Strains on the Volatile Profile of Apulian Wines
Source: Microorganisms. 2020 Apr 26;8(5):628. doi: 10.3390/microorganisms8050628 (PMC7285007; doi:10.3390/microorganisms8050628)
Supplement: Supplementary file 1 [file microorganisms-08-00628-s001.zip › Table S2.docx]

Table S2: Main oenological and technological properties determined in *C. zemplinina* strains

|  | H_2_S* | amino acid decarboxylation | | | | | | |
| --- | --- | --- | --- | --- | --- | --- | --- | --- |
|  |  | His | Tyr | Phe | Trp | Lys | Leu | Arg |
| 1NC1 | + | - | - | - | - | - | - | - |
| 7NC1 | + | - | - | - | - | - | - | - |
| 35NC1 | + | - | - | - | - | - | - | + |
| 19NC1 | ++ | - | - | - | - | - | - | - |
| 31NC1 | ++ | - | - | - | - | - | - | - |
| 19PR2 | - | - | - | - | - | - | - | - |
| 3NC1 | ++ | - | - | - | - | - | - | - |
| 4PR2 | ++ | - | - | - | - | - | - | - |
| 9PR2 | ++ | - | - | - | - | - | - | - |
| 23PR2 | - | - | - | - | - | - | - | - |
| 21NT1 | ++ | - | - | - | - | - | - | - |
| 20NT1 | - | - | - | - | - | - | - | - |
| 5PR1 | + | - | - | - | - | - | - | - |
| 15PR1 | + | - | - | - | - | - | - | - |
| 19PR1 | + | - | - | - | - | - | - | - |
| 18PR1 | + | - | - | - | - | - | - | + |
| 2T29 | + | - | - | - | - | - | - | - |
| 2T21 | + | - | - | - | - | - | - | - |
| 3T36 | + | - | - | - | - | - | - | - |
| 3T16 | + | - | - | - | - | - | - | - |
| 3KUT15 | + | - | - | - | - | - | - | - |
| 3KUT2 | + | - | - | - | - | - | - | - |
| 3KUT21 | + | - | - | - | - | - | - | - |
| 3KUT7 | + | - | - | - | - | - | - | - |
| 3TOR19 | + | - | - | - | - | - | - | - |
| 3TOR2 | + | - | - | - | - | - | - | - |
| 3TOR18 | + | - | - | - | - | - | - | - |
| FG6 | + | - | + | - | - | - | - | - |
| FG19 | + | - | - | - | - | - | - | - |
| FG21 | + | - | - | - | - | - | - | - |
| FG24 | + | - | - | - | - | - | - | - |
| FG27 | + | - | + | - | - | - | - | - |
| RT44 | - | - | - | - | - | - | - | - |
| IT8795 | - | - | - | - | - | - | - | - |
| 1KUT24 | +++ | + | + | + | + | + | + | + |

H_2_S and foam production: absent (¡); low (+), high (++), very high (+++)
