# Supplementary figures and images for: New Insights into the Oenological Significance of Candida zemplinina: Impact of Selected Autochthonous Strains on the Volatile Profile of Apulian Wines
Source: Microorganisms. 2020 Apr 26;8(5):628. doi: 10.3390/microorganisms8050628 (PMC7285007; doi:10.3390/microorganisms8050628)

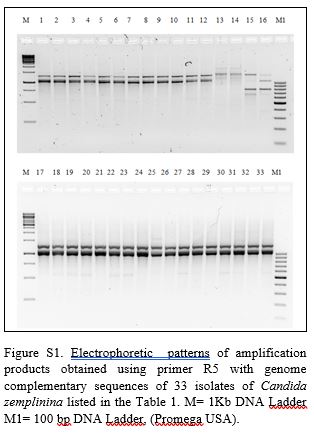

Supplement: Supplementary file 1 [file microorganisms-08-00628-s001.zip › Fig. S1.JPG]

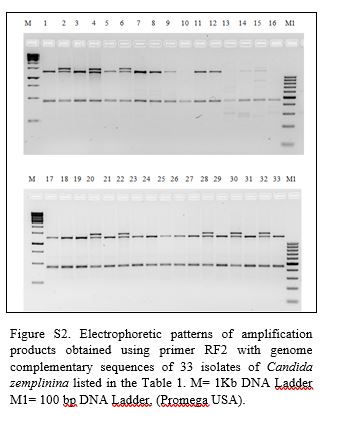

Supplement: Supplementary file 1 [file microorganisms-08-00628-s001.zip › Fig. S2.JPG]

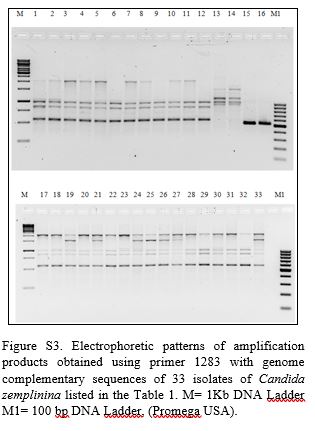

Supplement: Supplementary file 1 [file microorganisms-08-00628-s001.zip › Fig. S3.JPG]

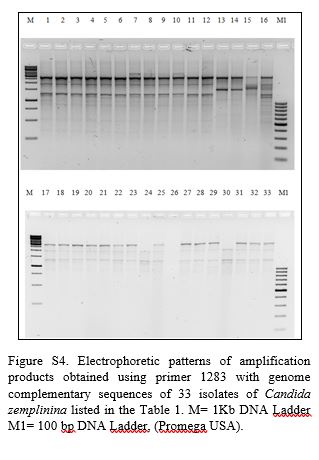

Supplement: Supplementary file 1 [file microorganisms-08-00628-s001.zip › Fig. S4.JPG]

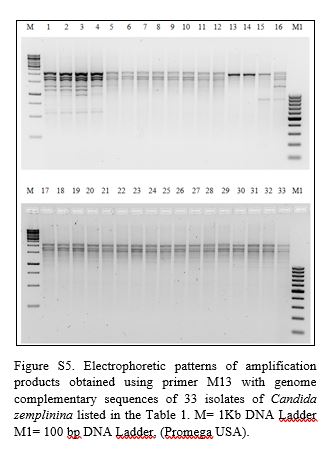

Supplement: Supplementary file 1 [file microorganisms-08-00628-s001.zip › Fig. S5.JPG]

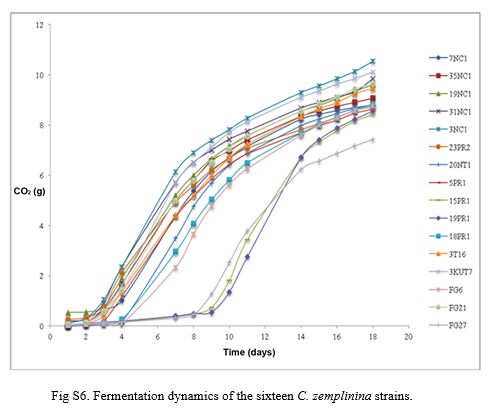

Supplement: Supplementary file 1 [file microorganisms-08-00628-s001.zip › Fig. S6.JPG]
